# Elevated Blood Lead Levels in Infants and Mothers in Benin and Potential Sources of Exposure

**DOI:** 10.3390/ijerph13030316

**Published:** 2016-03-11

**Authors:** Florence Bodeau-Livinec, Philippe Glorennec, Michel Cot, Pierre Dumas, Séverine Durand, Achille Massougbodji, Pierre Ayotte, Barbara Le Bot

**Affiliations:** 1 Ecole des Hautes Etudes en Santé Publique, 35043 Rennes, France; Philippe.glorennec@ehesp.fr (P.G.); Severine.Durand@ehesp.fr (S.D.); massougbodjiachille@yahoo.fr (A.M.); Barbara.LeBot@ehesp.fr (B.L.B.); 2 Inserm UMR 1153 Obstetrical, Perinatal and Pediatric Epidemiology Research Team (Epopé), Center for Epidemiology and Statistics Sorbonne Paris Cité, DHU Risks in Pregnancy, Paris Descartes University, 75014 Paris, France; 3 Inserm UMR 1085, Institut de Recherche en Santé, Environnement et Travail (IRSET), 35000 Rennes, France; 4 Institut de Recherche pour le Développement (IRD), Mère et Enfant Face aux Infections Tropicales, 75006 Paris, France; michel.cot@ird.fr; 5 Faculté des Sciences Pharmaceutiques et Biologiques, Sorbonne Paris Cité, Université Paris Descartes, 75006 Paris, France; 6 Laboratoire de Toxicologie, Institut National de Santé Publique du Québec, Québec, QC G1V 5B3, Canada; pierre.dumas@inspq.qc.ca (P.D.); pierre.ayotte@inspq.qc.ca (P.A.); 7 Faculté des Sciences de la Santé, Université d’Abomey-Calavi, Cotonou, Bénin; 8 Axe Santé des Populations et Pratiques Optimales en Santé, Centre de Recherche du CHU de Québec, Québec, QC G1V 5B3, Canada

**Keywords:** lead exposure, sources, child, sub-Saharan Africa, Lead Isotopic Ratios

## Abstract

Lead in childhood is well known to be associated with poor neurodevelopment. As part of a study on maternal anemia and offspring neurodevelopment, we analyzed blood lead level (BLL) with no prior knowledge of lead exposure in 225 mothers and 685 offspring 1 to 2 years old from Allada, a semi-rural area in Benin, sub-Saharan Africa, between May 2011 and May 2013. Blood samples were analyzed by inductively coupled plasma mass spectrometry. Environmental assessments in households and isotopic ratio measurements were performed for eight children with BLL > 100 µg/L. High lead levels (BLL > 50 µg/L) were found in 44% of mothers and 58% of children. The median BLL was 55.1 (interquartile range 39.2–85.0) and 46.6 (36.5–60.1) µg/L, respectively. Maternal BLL was associated with offspring’s consumption of piped water and animals killed by ammunition. Children’s BLL was associated with presence of paint chips in the house and consumption of animals killed by ammunition. In this population, with 98% of children still breastfed, children’s BLL was highly associated with maternal BLL on multivariate analyses. Environmental measures and isotopic ratios supported these findings. Offspring may be highly exposed to lead *in utero* and probably via breastfeeding in addition to lead paint exposure.

## 1. Introduction

Lead is well known to have detrimental effects on human health, including nervous, renal, cardiovascular, reproductive, immune and hematologic systems. In children, both postnatal and prenatal exposure to lead is associated with poor child neurodevelopment. Studies of postnatal exposure showed evidence of long-term effects on childhood intelligence quotient [1] as well as attention and inhibition [2,3]. Mechanisms may be related to impaired prefrontal cortex, the competition of lead with calcium, altered neuronal transmission, or interactions with thyroid hormones. Even very low blood lead level (BLL) may have adverse consequences [4]. As a consequence, the US Centers for Disease Control and Prevention recently revised the cut-off for elevated BLL from 100 µg/L to a level of concern, 50 µg/L [5].

A recent literature review of BLL in children <6 years old in sub-Saharan Africa (SSA) found only nine studies with data collection between 1991 and 2010 [6]. One of the main sources of lead was leaded gasoline [7], but most sub-Saharan countries began using unleaded gasoline in 2004–2005 (United Nations Environmental Programme 2011). In addition to prenatal exposure, reported potential sources of lead in SSA include piped water, leaded paint, medicinal products, battery smelter, eye cosmetics, clay, dust and metal mining [6,8]. 

Moreover, BLL may be increased with iron deficiency (ID) because lead and iron share common pathways for intestinal absorption [9]. ID is highly prevalent in pregnant women and children in SSA [10]. ID is also well known to affect neurodevelopment. Both BLL and ID may interact in child development and behavior (*i.e*., children with ID may be more susceptible to the detrimental effect of lead [9]). Therefore, as part of a study of the impact of maternal anemia during pregnancy on offspring neurodevelopment, we assessed BLL as a potential confounder and/or modifier in children 1 to 2 years old in a semi-rural area in Benin. We found elevated BLL. The aim of this paper was to report BLL in children 1 to 2 years old and mothers in a semi-rural area in Benin and to discuss the likely sources of exposure. 

## 2. Material and Methods

As the investigation of elevated BLL was not initially planned but due to incidental discovery, the methods section contains successive steps.

Our study sample included singletons born to pregnant women enrolled in the “*Malaria in Pregnancy Preventive Alternative Drugs*” (*MiPPAD*) clinical trial (NCT00811421) comparing two intermittent preventive treatments of malaria in pregnancy [11]. The study was conducted in three health centers in the Allada district (Sekou, Allada and Attogon), South Benin. All surviving infants of recruited pregnant women were invited to undergo neurocognitive assessment in the TOVI study (Fon language: *Tovi* means child from the country) when the child was 12 months old [12]. From May 2011 to May 2013, blood samples were taken from 685 children 12 to 24 months old. Children with BLL > 250 µg/L were invited to be assessed for free a second time for BLL (9 of 14 reassessed). Mothers were not initially planned to be assessed for lead. After the first 50 BLL assessments, we decided to assess as many mothers as possible when offspring were 18 or 24 months through the TOLIMMUNPAL project, which resulted in 227 blood samples from mothers. 

Information on socioeconomic status and home environment were gathered during a home visit when the child was 12 months old. This questionnaire administered by a nurse or a psychologist included information on potential sources of lead including presence of paint and paint chips in the household, maternal and paternal occupation classified by risk of lead exposure [13], breastfeeding and sociodemographic characteristics. A family wealth scale involved a scoring instrument incorporating a checklist of material possessions (radio, television, bike, motorbike, and car), keeping cows, and access to electricity. Following the first results of BLL, we included a second questionnaire on potential sources of lead including sources of water for the child, pica behavior (ingestion of substances; here white and green clay) in children and in mothers during pregnancy, gasoline stored at the home, cooking and eating utensils [14], the child’s consumption of meat from animals killed by lead ammunition, maternal use of cosmetics, activities in the house or neighborhood, and number of hours the child played outside the house. This questionnaire was completed for 623 children: 53% when the child was > 12 months old because of the delay in implementing this second questionnaire (median 20.1 months, range 11.3–35.1). 

We collected 8 ml venous blood from each participant, 4 mL in a tube containing dipotassium EDTA and 4 mL in an iron-free dry tube. Blood samples were analyzed at the Centre de Toxicologie, Institut National de Santé Publique du Québec (Québec, Canada), by inductively coupled plasma mass spectrometry (ICP-MS; Perkin Elmer Sciex Elan DRC II ICP-MS instrument) before 20-fold dilution in amonia 0.5% v/v and 0.1% v/v surfactant Triton-X. The limit of detection for blood analysis was 0.2 µg/L. 

Furthermore, to investigate exposure pathways, we visited eight households for environmental and food assessments. Households were selected if the child’s BLL was > 100 µg/L (very high BLL) and a sufficient amount of blood was available to perform lead isotope ratio (LIR) measurements. The LIR is the ratio of abundance of lead with different atomic weight because of a different neutron number. LIR can provide information from lead contamination origins because the isotopic signature varies by the age of the original ore. The concentration of lead in soil, water, gasoline, dishes, food and paint, when present, was determined in each household. Ammunition for animal hunting was purchased from a local market. All samples were analyzed by ICP-MS (Agilent technologies 7500ce ICP-MS instrument) at a school of public health (EHESP, Rennes, France). For water, the limit of quantification (LOQ) was 1 µg/L. Before ICP-MS analysis, food, soil and gasoline were mineralized with a mixture of nitric and hydrochloric acid (1/3 HNO_3_ and 2/3 HCl) by microwave (Multiprep 41, Milestone) and dust by a graphite block digestion system (Digiprep, SCP Science). The LOQ was 0.1 mg/kg for food, 0.1 mg/kg for soil, 0.5 mg/L for gasoline and 2 µg/m^2^ for dust. Dishes were soaked in a solution of acetic acid (4%) for 24 h before analysis, and the LOQ was 2 µg/L. 

The LIR in children’s blood and possible sources of lead was determined by the concentration of lead in each source for a given child. The LIR was determined in the most concentrated sample of each exposure media by using quadrupole ICP-MS (Agilent technologies 7500ce ICP-MS instrument) with environmental samples at EHESP. The mass bias was corrected with a certified reference material (Common Lead Isotopic Standard, SRM 981, NIST) with the standard bracketing technique [15]. 

Lead stable isotope ratios in blood were established with low-resolution quadropole ICP-MS (Perkin Elmer NexION 300S instrument) after proper dilution to an obtained final lead concentration of 5 µg/L in a 0.5% (v/v) ammonia solution with 0.1% v/v surfactant Triton-X. Instrumental isotopic ratio responses were calibrated with certified reference materiel NIST SRM 981 and controlled with SRM NIST 982.

Intercalibrated LIR was calculated by the two laboratories and involved two aqueous leaded samples. Each of the two laboratories used its own method for determining mass correction with the standard SRM 981 and correction of blanks. The results were comparable, except for LIR containing 204.

Interpretation was based on the proximity of graphical isotopic ratios between blood and potential sources, accounting for measurement accuracy (a source was considered compatible with blood with recovery between the error bars of the source and blood) [16] and assessed on isotopic ratios 208/207 *vs.* 207/206.

In statistical analyses, BLL was log-transformed for normal distribution (natural logarithm). We first described BLL in children and mothers, potential sources of lead, and sociodemographic characteristics of the study population. Second, we performed univariate analysis to assess the crude association between the potential sources of lead in both children and mothers, sociodemographic characteristics, and BLL. Third, we conducted multivariate analyses in three steps for children with logBLL and BLL >50 µg/L as dependent variables: model 1 included potential sources of lead identified on univariate analyses with *p* < 0.05; model 2 included socioeconomic factors in addition to sources of lead; model 3 included maternal BLL in addition to the former variables. The first two models were also used to analyze maternal BLL. 

Student *t* test and chi-square test were used to compare means and proportions, respectively. Multiple linear regression and logistic regression were used for continuous and binary outcomes, respectively. Statistical significance was defined as *p* < 0.05. Statistical analyses involved use of SAS 9.3.

### Ethics

The study was approved by the institutional review boards of the University of Abomey-Calavi in Benin and New York University in the United States (IRB#09-1253). At recruitment, we obtained informed consent from all pregnant women and guardians of children who participated in this study. 

## 3. Results

Overall, 58% of children and 44% of mothers showed elevated BLL (BLL > 50 µg/L; median BLL 55.1 [interquartile range 39.2–85.0] and 46.6 [36.5–60.1] µg/L, respectively) (Table 1). Child and maternal BLLs were correlated: r = 0.30 (*p* < 0.001).

Many children had eaten soil and most were drinking piped water; few children were living in households with painted walls or had activities that may involve use of lead (Table 2). Overall, 39% were eating meat from animals killed by ammunition. Crude associations between potential sources of lead in children and mothers and BLL are in Table 2.

On multivariate analysis, paint in the household and eating animals killed by ammunition were significantly associated with children’s logBLL after adjustment for socioeconomic factors (Table 3). In model 3, maternal BLL and paint chips were the only significant predictors of logBLL. The estimate for eating animals killed by ammunition decreased from 0.11 in model 2 to 0.04 in model 3 (Table 3). For mothers, their offspring’s consumption of piped water and animals killed by ammunition were associated with elevated BLL and with logBLL.

Because almost all children were still breastfed at 12 months (98%), breastfeeding was not included in the model. However, the median BLL was 47.5 µg/L (*n* = 13) for children not breastfed compared with 55.7 µg/L (*n* = 668) in children breastfed.

For eight children with BLL > 100 µg/L, we tested potential sources of lead exposure (Table 4). Dust lead loadings are difficult to interpret because lead loading highly depends on the amount of dust collected, which was high in samples collected on clay soils. Ammunition was composed of only lead, with no lead in samples of gasoline. The cooking dish in one household and paint in another household had high lead contents.

**Figure 1:**
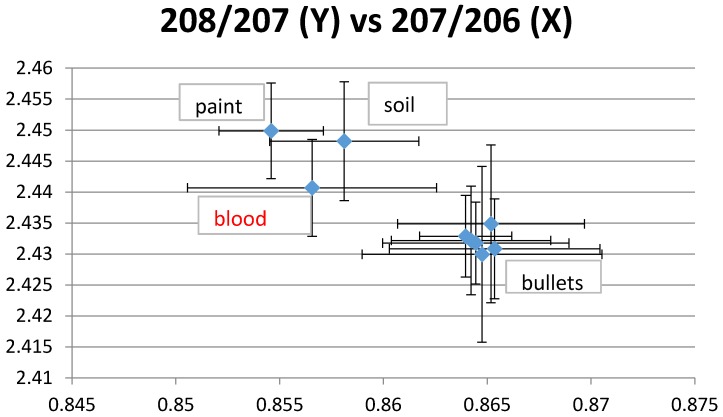
Example 1 of lead isotope ratios (LIRs). The plotted blood LIRs with their confidence intervals (±2 SD) intercept those of paint, soil and bullets.

**Figure 2:**
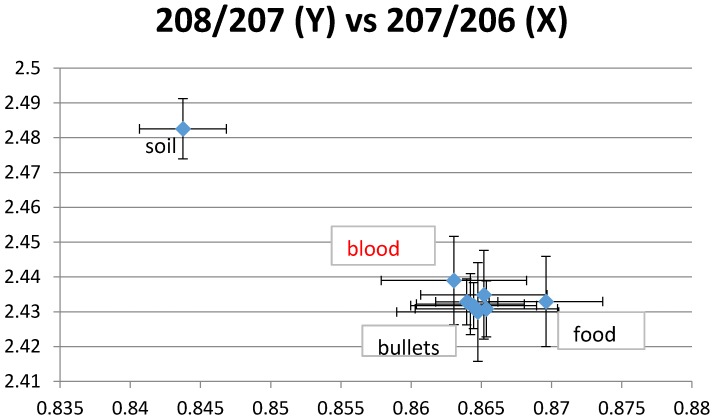
Example 2 of LIRs. The plotted blood LIRs with their confidence intervals (± 2 SD) intercept those of food and bullets but not soil.

LIRs for ammunition were all very close, which indicates a unique isotopic signature. Blood LIRs were compatible with ammunition isotopes for 6 of 8 children, including three who did not eat animals killed by ammunition but who were still breastfed at the time of the assessment. Ammunition, paint and dust in the house were possible sources of lead for another child (Figure 1). In addition to ammunition, house dust was a possible source of exposure for two children and food for one child (Figure 2). For two children, ammunition was the only possible tested source of lead. Blood LIRs were not compatible with any measured potential source for two infants.

## 4. Discussion

With no prior knowledge, we found elevated BLL in children in Benin, SSA. Multiple potential sources of lead were identified, including consumption of meat from animals killed by ammunition, piped water, paint, and soil. To our knowledge, this is the first time eating animals killed by ammunition has been identified as a potential source of lead in SSA children. 

Our study is one of the few describing BLL in children at one year old and in mothers and one of the largest of studies performed in SSA. An important strength of our study is the use of several and complementary approaches, including blood measures, questionnaires, lead source concentration measurements and isotopes in both blood and environment matrices.

According to a literature review of BLLs in SSA children < 6 years old, the prevalence of BLL>100 µg/L ranged from 7.0% to 70.9% [6] as compared with 16.5% in our study. In 6 of 9 studies, at least half of the children had BLL ≥100 µg/L. The mean was 162 µg/L as compared with 72 µg/L in our children. Several reasons may explain these discrepancies. First, most of the children in our study were assessed at age 12 months, hence at a younger age than in previous studies. As found in our results and in the literature, BLL increases with age in early childhood [17]. Second, our study was performed in a semi-rural area. Urban settings seem more exposed to lead than rural settings. Third, data collection in all studies occurred before 2010 and in three studies before 2000. Leaded gasoline is an important source of lead exposure [6,18], but we did not find lead in gasoline in our samples nor a statistical association. This study also suggests the possible involvement of other well-known sources of lead described in other SSA countries including dust in soil, leaded paint [6,8,19] and piped water [20]. Contrary to other studies of SSA [6,8], leaded gasoline, eye cosmetics, clay, and battery smelter were not suspected to be a source of lead in our sample according to the statistical analyses or environmental assessments. Finally, this setting did not involve metal mining as in other studies, with higher prevalence of elevated BLL [6]. 

Leaded gasoline was banned in 2005 in Benin. Indeed, leaded gasoline was not a current source of exposure, as shown in our survey and in environmental measures. Mothers were probably exposed to leaded gasoline in the past. In addition to this baseline exposure, we found an association between maternal BLL and offspring consumption of piped water and animals killed by ammunition. 

The correlation we found between maternal and children’s BLL was probably due to a high proportion of children’s BLL acquired both *in utero* and potentially post-natally through breastfeeding. Lead stored in bones is released during pregnancy and the breastfeeding period, thereby increasing the BLL in mothers [21]. Breastfeeding is a known possible source of lead [22] and probably important in our study, given the high prevalence of breastfeeding in children 12 months old (98%), although breastmilk could not be analyzed to confirm this hypothesis. Mothers showed a lower BLL than their offspring, probably because of the higher absorption of lead in children than adults [23]. 

A major finding of our study is the identification of lead fragments in hunted animals as a source of lead exposure, as was found in other parts of the world [24] but not to our knowledge in SSA. Almost all children (99%) at age 12 months were sharing the family meals. About 39% of children were eating meat from animals killed by lead shots. These animals included rabbits and small poultry. This finding is important because recommendations may be formulated for populations to decrease BLL.

The elevated BLL we observed may also be explained in part by the high level of anemia and ID in our study population. Indeed, the prevalence of anemia was 66% in children aged 12 months. ID may increase lead absorption probably by the regulation of the apical divalent metal transporter-1 [25]. According to the WHO, 47% of children <5 years old worldwide are anemic and may therefore be more susceptible to lead exposure [26]. The consequences of lead on children’s health have been less studied in SSA than in other parts of the world. Further research is warranted because risk factors differ, for example in terms of malaria [27] and malnutrition.

Because elevated BLL was unknown, this complementary research project was set up in an emergency. A limitation of our study is the lack of information on consumption by mothers of animals killed by ammunition, as this was only collected in children. In addition, the complementary questionnaire on potential sources of lead was completed later than the actual time of blood sampling for half of the children. Therefore, the prevalence of consumption of animals killed by ammunition may be overestimated but may be closer to maternal consumption and may explain the association with maternal BLL. Because only 2 cooking dishes and only one meal were assessed in each household, we cannot exclude that children could be exposed to lead through this pathway. 

## 5. Conclusions

We assessed BLL in infants as part of a study on neurocognitive development with no prior knowledge of the level of BLL and potential exposure to lead. Our striking results show that environmental threats are underestimated in low-income countries. In these settings, with high prevalence of malaria and malnutrition and important consequences in terms of mortality and morbidity, environmental concerns are often seen as secondary. Further research and public health investigations should be conducted in these countries by monitoring BLL in different settings (urban and rural), especially in children and pregnant women. These studies should include representative samples, larger numbers of households, comprehensive measurements of LIR and specific studies on infant hand-to-mouth behaviors. Indeed, in this hot and humid climate, children’s hands may be likely covered with dust and therefore their BLL may be higher than in children from high-income countries for a given concentration of lead in dust. Assessments of BLL and treatments with elevated BLL are not often available as is the case in Benin. There is a need for capacity building in environmental assessments, treatments and research in this field in SSA. BLL seems to have decreased in high-income countries but may be a growing concern in less developed countries. For example, the prevalence of elevated BLL in the United States in 2011 was 0.6% as compared with 57.8% in our study [28]. Given the neurotoxicity of lead, this finding highlights what Grandjean and Landrigan called the “global, silent pandemic of neurodevelopmental toxicity” [4].

## Figures and Tables

**Table 1:** Blood lead level (BLL, µg/L) in children 12 to 24 months old and mothers at 18 months post-partum in Allada, Benin.

Blood Lead Level	Children *N* = 685	Mothers *N* = 227
Mean ± SD	72.2 (60.8)	51.4 (22.3)
Median	55.1	46.6
Minimum	8.3	22.8
Maximum	630.0	202.0
1st quartile	39.2	36.5
3rd quartile	85.0	60.1
% over guideline value, % (n)		
BLL>100 (µg/L)	16.4 (112)	2.6 (6)
BLL>50 (µg/L) *	57.8 (396)	43.6 (99)

* BLL > 50 includes children with BLL > 100 µg/L.

**Table 2:** Univariate analysis of potential sources of lead (Pb) and BLL in children and mothers.

Potential Sources of Pb	In Children			In Mothers	
	*n* (%)	Increment in LogBLL Compared to Referent Group (95% CI)	Crude OR for BLL>50 µg/L (95% CI)	Increment in LogBLL Compared to Referent Group (95% CI)	Crude OR for BLL>50 µg/L (95% CI)
Child has ever eaten soil	338 (56.1)	0.01 (−0.08,0.11)	0.78 (0.56,1.08)	0.05 (−0.06,0.15)	
Child drinking any piped water	499 (82.8)	0.08 (−0.04,0.20)	0.95 (0.62,1.47)	0.16 (0.04,0.28) *	2.42 (1.19,4.89) *
Child drinking any well water	333 (55.2)	0.07 (−0.03,0.16)	1.08 (0.78,1.49)	−0.10 (−0.20, −0.01) *	0.59 (0.34,1.02)
House in mud	413 (61.0)	0.07 (−0.03,0.16)	1.25 (0.92,1.71)	0.03 (−0.08,0.13)	1.28 (0.73,2.25)
Painted walls in household	96 (14.1)	0.07 (−0.05,0.20)	1.14 (0.73,1.77)	−0.03 (−0.17,0.12)	0.68 (0.30,1.56)
Presence of paint chips in household	35 (5.8)	0.23 (0.03,0.42) *	1.26 (0.62,2.54)	−0.04 (−0.25,0.18)	0.95 (0.29,3.10)
Gasoline stored at home	375 (62.2)	−0.11 (−0.20,-0.01)	0.70 (0.50,0.97) *	−0.07 (−0.17,0.03)	0.94 (0.53,1.66)
Cooking utensils with recycled material	603 (100.0)	−	−	−	−
Metal cooking utensils	360 (59.7)	−0.05 (−0.15,0.04)	0.76 (0.54,1.06)	−0.05 (−0.15,0.05)	0.94 (0.55,1.62)
Mud cooking utensils	272 (45.1)	0.04 (−0.05,0.13)	1.00 (0.72,1.38)	−0.11 (−0.11,0.9)	0.86 (0.50,1.49)
Enamel eating utensils	581 (96.4)				
Pottery eating utensils	55 (9.1)	0.06 (−0.10,0.22)	1.21 (0.68,2.13)	−0.05 (−0.22,0.12)	1.23 (0.48,3.15)
Child eats animals killed by ammunition	234 (38.8)	0.11 (0.01,0.20) *	1.50 (1.07,2.09) *	0.09 (−0.00,0.19)	1.81 (1.04,3.14) *
Maternal consumption of clay during pregnancy	180 (26.3)				
Green clay (kalaba)	142 (23.5)	−0.00 (−0.11,0.11)	1.08 (0.74,1.58)	0.07 (−0.05,0.19)	1.15 (0.59,2.23)
White clay (kaolin)	39 (10.9)	−0.05 (−0.25,0.15)	0.76 (0.39,1.49)	−0.10 (−0.27,0.07)	0.65 (0.24,1.79)
Maternal use of eye cosmetics	113 (18.7)	0.01 (−0.11,0.13)	0.95 (0.63,1.43)	−0.11 (−0.25,0.04)	0.63 (0.28,1.42)
Breastfeeding at 12 months old	668 (98.1)				
Paternal high or moderate risk of occupational Pb exposure	132 (19.7)	−0.01 (−0.13,0.10)	0.81 (0.55,1.19)	0.10 (−0.03,0.23)	1.26 (0.62,2.56)
Activity in child’s house or neighborhood:					
Metal smelter	17 (2.8)				
Battery recycling/storage	3 (0.5)				
Radiator repair	3 (0.5)				
Metal recycling/storage	4 (0.7)				
Lead solder	3 (0.5)				
Vehicle repair (car, motorcycle)	32 (5.3)				
Manufacturing of ammunition, metal fish baits or metal objects	1 (0.2)				
Sociodemographic characteristics:					
Child’s age at blood sampling (≥17 months)	74 (10.9)	0.17 (0.03,0.32) *	1.37 (0.85,2.20)		
Child’s gender (male)	348 (51.1)	–0.02 (–0.10,0.07)	1.08 (0.80,1.47)		
Time playing outside:					
<2 h/day	27 (4.48)	0	1		
2–6 h/day	254 (42.1)	0.05 (−0.18,0.28)	1.48 (0.67,3.28)		
>6 h/day	322 (53.4)	0.09 (−0.14,0.32)	1.51 (0.69,3.32)		
Wealth score (median ± SD)	5 (2.76)				
Collective housing	100 (14.8)	−0.15 (−0.28,−0.02) *	1.39 (0.91,2.12)	−0.09 (−0.23,0.04)	1.65 (0.76,3.59)
Maternal age (years)		−0.05 (−0.13,−0.03)	0.87 (0.67,1.14)	−0.01 (−0.10,0.07)	1.13 (0.71,1.80)
Maternal education ≥ high school	53 (7.8)	0.11 (−0.06,0.27)	1.22 (0.68,2.17)	−0.04 (−0.21,0.13)	1.07 (0.43,2.70)
Working mother	628 (92.2)	−0.18 (−0.35,−0.02) *	0.75 (0.42,1.34)	−0.17 (−0.39,0.04)	0.37 (0.11,1.26)
Married	673 (99.4)				
Polygamous	247 (36.7)	0.01 (−0.08,0.11)	1.20 (0.87,1.65)	0.02 (−0.08,0.12)	1.46 (0.85,2.53)
Maternity ward					
Sekou	421 (61.8)	0	1		
Attogon	200 (29.4)	0.08 (−0.02,0.18)	0.87 (0.50,1.52)	0	1
Allada	60 (8.8)	−0.01 (−0.17,0.15)	1.17 (0.84,1.65)	−0.01 (−0.13,0.10)	0.87 (0.47,1.61)

* *p* < 0.05.

**Table 3:** Multivariate analysis of association between potential sources of lead and BLL after adjustment for other sources of lead, socioeconomic status and maternal BLL.

	In Children		In Mothers	
	Increment in LogBLL Compared to Referent Group (95% CI)	Adjusted OR for BLL>50 µg/L (95% CI)	Increment in LogBLL Compared to Referent Group (95% CI)	Adjusted OR for BLL>50 µg/L (95% CI)
Model 1 ^1^	(*n* = 601)	(*n* = 601)	(*n* = 215)	(*n* = 215)
Presence of paint chips in household	0.21 (0.01,0.41) *	1.22 (0.60,2.50)	−0.09 (−0.31,0.12)	0.69 (0.21,2.32)
Child eats animals killed by ammunition	0.12 (0.02,0.21) *	1.49 (1.06,2.09) *	0.10 (0.01,0.20) *	1.92 (1.09,3.38) *
Piped water	0.08 (−0.05,0.20)	0.98 (0.64,1.51)	0.17 (0.05,0.29) *	2.60 (1.27,5.36) *
Model 2 ^2^	(*n* = 598)	(*n* = 598)	(*n* = 212)	(*n* = 212)
Presence of paint chips in household	0.21 (0.01,0.40) *	1.29 (0.62,2.67)	−0.13 (−0.35,0.08)	0.56 (0.16,1.97)
Child eats animals killed by ammunition	0.11 (0.01,0.20) *	1.45 (1.03,2.05) *	0.10 (−0.00,0.19)	1.86 (1.05,3.31) *
Piped water	0.10 (−0.02,0.23)	1.09 (0.69,1.72)	0.19 (0.07,0.32) *	2.56 (1.20,5.46) *
Child age at blood sampling (≥17 months)	0.02 (−0.00,0.05)	1.50 (0.87,2.59)		
Type of housing	0.12 (−0.02,0.25)	1.07 (0.66,1.72)	0.08 (−0.06,0.22)	1.72 (0.74,3.99)
Gasoline stored at home	−0.09 (−0.20,0.03)	0.77 (0.52,1.12)	−0.05 (−0.17,0.06)	0.87 (0.44,1.70)
Wealth score	−0.01 (−0.03,0.01)	0.97 (0.90,1.04)	−0.01 (−0.03,0.01)	1.03 (0.91,1.16)
Maternal occupation	−0.21 (−0.39,−0.04) *	0.67 (0.35,1.29)	−0.18 (−0.40,0.04)	0.38 (0.10,1.43)
Model 3 ^3^	(*n* = 193)	(*n* = 193)		
Presence of paint chips in household	0.49 (0.13,0.86) *	0.69 (0.18,2.57)		
Child eats animals killed by ammunition	0.04 (−0.13:0.21)	1.27 (0.69,2.34)		
Piped water	−0.01 (−0.21,0.20)	0.88 (0.42,1.86)		
Mother BLL	0.01 (0.00,0.01) *	1.02 (1.00,1.03)		
Child age (≥17 months)	0.03 (−0.00,0.06)	1.32 (0.68,2.58)		
Type of housing	−0.10 (−0.34,0.14)	0.76 (0.32,1.79)		
Gasoline stored at home	0.00 (−0.21,0.21)	0.75 (0.37,1.53)		
Wealth score	0.02 (−0.02,0.05)	1.02 (0.90,1.16)		
Maternal occupation	−0.21 (−0.57,0.14)	0.61 (0.15,2.52)		

* *p* < 0.05. ^1^ Model 1 included only potential sources of lead (presence of paint chips in household, child eats animals killed by ammunition and piped water); ^2^ Model 2 included potential sources of lead (presence of paint chips in household, child eats animals killed by ammunition and piped water) and socioeconomic factors (child age, type of housing, gasoline stored at home, wealth score and maternal occupation); ^3^ Model 3 included potential sources of lead (presence of paint chips in household, child eats animals killed by ammunition and piped water), maternal lead and socioeconomic factors (child age, type of housing, gasoline stored at home, wealth score and maternal occupation).

**Table 4:** Lead concentrations in potential sources for children with BLL>100.

Child No.	BLL in Infants (µg/L)	Dust (µg/m^2^)	Soil (mg/kg)	Drinking Water (µg/L)	Cooking Dish (µg/L)	Paint (mg/kg)	Food (µg/kg)	Child Eats Animals Killed by Ammunition	Paint Inside House	Still Breastfed
1	110	*1735*	25	<LOQ *			111	*Yes*	No	Yes
2	135	*189*	3	<LOQ				*Yes*	No	Yes
3	99	*1640*	34	1.1			411	*Yes*	Yes	Yes
4	109	9	4	2.3			< LOQ	*No*	Yes	Yes
5	209	107	33	<LOQ		*7020*	94	*Yes*	No	Yes
6	136	19	4	<LOQ			< LOQ	*Yes*	No	Yes
7	105	*294*	48	<LOQ			< LOQ	*Yes*	No	Yes
8	113	32	3	*5.4*	*4952*			*No*	No	Yes

Italic: IR measured—Bold: IR compatible with blood; * The LOQs were 1 µg/L for water, 0.1 mg/kg for food, 0.1 mg/kg for soil, 0.5 mg/L for gasoline and 2 µg/m^2^ for dust.
